# Persistent reduction of *Bifidobacterium longum* in the infant gut microbiome in the first year of age following *intrapartum* penicillin prophylaxis for maternal GBS colonization

**DOI:** 10.3389/fimmu.2025.1540979

**Published:** 2025-05-15

**Authors:** Jana Lucia Teuscher, Mariia Lupatsii, Simon Graspeuntner, Sinje Jonassen, Arne Bringewatt, Egbert Herting, Guido Stichtenoth, Verena Bossung, Jan Rupp, Christoph Härtel, Martin Demmert

**Affiliations:** ^1^ Clinic for Pediatric and Adolescent Medicine, University Hospital Schleswig-Holstein, Lübeck, Germany; ^2^ Department for Infectious Diseases and Microbiology, University Hospital Schleswig-Holstein, Lübeck, Germany; ^3^ German Center for Infection Research, Partner Site Hamburg-Lübeck-Borstel-Riems, Lübeck, Germany; ^4^ Medical Clinic III, University Hospital Schleswig-Holstein, Lübeck, Germany; ^5^ Clinic for Gynecology and Obstetrics, University Hospital Schleswig-Holstein, Lübeck, Germany; ^6^ Pediatric Clinic and Policlinic, University Hospital Würzburg, Würzburg, Germany

**Keywords:** *intrapartum* antibiotic prophylaxis (IAP), group B Streptococcus, early life microbiota, neonatal immunity, intestinal microbiome

## Abstract

**Introduction:**

Group B Streptococcus is a significant cause of early-onset disease in term newborns, with a global incidence of 0.41/1000 live births. Intrapartum antibiotic prophylaxis (IAP) has reduced EOD incidence by over 80%, but concerns exist about its impact on the neonatal gut microbiome and potential long-term health effects.

**Methods:**

This single center study examines the effects of IAP on the fecal infant microbiome in the first year of age and on the T cell phenotype in the first days after birth among 22 infants receiving IAP with penicillin due to maternal GBS colonization and 26 infants not exposed to IAP. The fecal microbiome was analyzed at birth, one month and one year of age through 16S rRNA gene sequencing. Additionally, a T cell phenotyping of peripheral blood was performed between the second and fifth day of age.

**Results:**

At one month, IAP exposed infants had a significantly lower relative abundance of Bifidobacterium longum in fecal samples, an effect which was sustained at one year. In IAP exposed infants we found a proinflammatory T-helper cell profile, characterized by higher IL-17A, RORgt, and TGF-b expression.

**Discussion:**

This study proposes a sustained impact of IAP on the neonatal microbiome and T cell repertoire.

## Introduction

Group B Streptococcus (GBS) remains the leading cause of early onset disease (EOD) in term newborns (1) (2). Globally, the incidence of early onset disease by GBS is estimated to be 0.41/1000 live births (3), contributing substantially to neonatal morbidity and mortality. For Germany, an incidence of 0.15/1000 live births was reported by a prospective active surveillance study covering the years 2009 and 2010 (4). Vertical transmission of GBS occurs during birth in 35-63% of colonized women, if no IAP was administered (5) (6) (7). Globally, Russell et al. estimated 18% of pregnant women to have evidence for GBS in rectovaginal cultures (8), while estimates for Germany vary between 16% and 21%. Rectovaginal colonization is usually asymptomatic but can be a cause of chorioamnionitis in pregnant women (9) (10) (11).

Since the introduction of *intrapartum* antibiotic prophylaxis (IAP) in the 1990s, the incidence of EOD by GBS has decreased by more than 80% in the United States (12) (13), while late-onset GBS risk remains largely unchanged (12). Noteworthy, early antibiotic exposure can be associated with several adverse sequelae, e.g. obesity and overweight (14) (15), asthma, eczema and allergy (15) and Crohns disease in children (16). Therefore, concerns have arisen about the potential impact of IAP on the neonatal gut microbiome. The gut microbiome exerts a variety of functions within the human organism, such as biosynthesis of vitamins (17) and short-chain fatty acids providing additional energy to the host and lowering the luminal pH (17) (18) (19) (20), (21), protection against pathogens (18) (21), and regulation of intestinal permeability (22) (23). Furthermore, the gut microbiome interacts with the immune system influencing for instance the T_reg_/T_H_17-balance (24) (25) (26). Alterations of the gut microbiome in early life have implications for immune-related diseases such as atopy and asthma (27) as well as autoimmune diseases (28) later in life. Numerous studies have investigated the impact of IAP in vaginally delivered term infants on neonatal gut microbiota with conflicting results (29–44). However, one consistent effect of IAP in vaginally delivered full-term infants is the reduction of *Bifidobacterium* and *Bacteroides*, whereas on phylum-level *Proteobacteria* are increased in gut microbiota within the first three months following IAP (45) (46) (47). A meta-analysis found a non-significant reduction in infant α-diversity following IAP exposure including studies with fecal sampling between one week and three months (48). However, only few studies have investigated this impact beyond the first weeks after birth. Immune-microbiome interactions following IAP have not yet been examined to our knowledge.

In this study, we investigated the impact of the IAP on the infant microbiome immediately after birth and during the first year of age, as well as concomitant alterations of the immunophenotype of T cells within the first days after birth. We focused on T cells, as adaptive immune system-alterations are well described in animals and humans following microbial pertubations (49) (27), possibly due to the close interactions between the gut microbiome and T_regs_ or T_H_17 cells (24) (25) (26).

## Methods

### Study population

Between May 2019 and October 2020 n = 48 (n = 22 GBS positive; n = 26 GBS unknown (5) or negative (21)) mothers and their newborns were recruited in a single center study at the University Hospital of Lübeck, Germany. Written informed consent was obtained. Inclusion criteria were: at least 37 completed weeks of gestation, maternal age ≥ 18 years, informed consent of both parents, singleton pregnancy and vaginal birth. Exclusion criteria were antibiotic therapy within 8 weeks before birth, maternal smoking, insulin dependent diabetes mellitus, clinical signs of amniotic infection (maternal fever > 37.8°C without other cause or maternal tachycardia > 120 beats per minute (bpm) or fetal tachycardia > 180 bpm or purulent and foul-smelling amniotic fluid/discharge or maternal leukocytosis > 15,000/µl) or penicillin allergy. The study was approved by the Ethics committee of the University of Lübeck (reference numbers 19-022 and 15-304).

### Stratification

Participants were assigned to one of two groups according to maternal GBS status. Mothers with cultural proof of rectovaginal GBS colonization – obtained between 35 and 37 weeks of gestation and cultured on selective media - received IAP according to national guidelines (5 million IU Benzylpenicillin from the onset of labor or rupture of membranes, followed by 2.5 million IU every 4h until birth intravenously (50)). Mothers without proof of rectovaginal GBS colonization (either negative culture or not tested and no risk factors for EOD by GBS) did not receive any *intrapartum* antibiotic prophylaxis, here referred to as “control group”.

### Sample collection

Rectal swabs were obtained from the mothers perinatally and after birth from the newborn. Meconium was collected, immediately stored at -20°C and timely transferred to -80°C. Cord blood was collected immediately after birth for assessment of penicillin concentrations. For the T cell panel, 100 µl EDTA-blood were obtained once between the second and fifth day after birth in a subset of 22 infants during routine blood sampling (sepsis workup or newborn screening) by venipuncture of a peripheral vein. At one month and one year of age, stool collection tubes and instructions were mailed to the families. Stool samples were then collected at home by the parents, stored in a freezer and picked up by study personnel. The samples were transported into the lab on ice and stored at -80°C. Health data (concerning e.g. nutrition, infections, body measurements, medication and diseases) were collected via a standardized questionnaire at birth, one month and one year by study personnel.

### DNA isolation

Samples were thawed at room temperature and approximately 100 mg of stool or 200 mg of meconium, were processed using the DNeasy PowerSoil Kit or DNeasy PowerSoil Pro Kit (Qiagen GmbH, Hilden, Germany) following the manufacturer´s instruction with some modifications: 20 µl Proteinase K were added to solution C1. For the DNA-extraction of meconium samples, DNA was washed twice with the C5-solution and eluted in 60 µl C6-solution. A negative extraction control was performed with each batch. Extracted DNA was stored at -20°C until subsequent use. For three children no meconium samples were available, therefore rectal swabs obtained immediately after birth were used for DNA-extraction and analysis in these three cases.

### 16S rRNA gene amplification, sequencing and bioinformatic processing

Partial sequences of the 16S rRNA gene in DNA samples were amplified using primers for V3/V4 hypervariable regions of the 16S rRNA gene (**Supplementary Table 1**) as described earlier (51). Polymerase chain reaction (PCR) was performed with the following parameters: 98°C for 30 seconds, 30 cycles with 98°C for 9 seconds, 55°C for 60 seconds and 72°C for 90 seconds. Meconium samples passed 35 PCR-cycles. In each PCR, a negative and a positive control were included, only PCRs with a clean negative control were subsequently used. Amplicons were run in gel electrophoresis and concentrations were estimated using Bio 1D software (Vilber Lourmat, Eberhardzell, Germany) against the 100 bp DNA ladder (Thermo Fischer Scientific, Waltham, USA). Equimolar amounts of each amplicon were pooled into a library and – after a run on an agarose gel – purified using the MiniElute Gel Extraction Kit (Qiagen GmbH, Hilden, Germany) and quantified with the NEBNExt Library Quant Kit (New England Biolabs, Ipswich, USA). The library was sequenced using the MiSeq platform (Illumina, San Diego, USA) and the MiSeq reagent Kit V3 for 600 cycles. PhiX library served as positive control. Negative extraction controls were incorporated to prove lack of contamination.

Fastq files were processed using mothur version 1.43.0 (mothur.org) (52) (53). Quality control steps included removal of sequences with homopolymers of more than 12 bases or sizes longer than 500 bp, removal of non-aligned sequences after alignment against EzBioCloud reference data base (54) and removal of chimeric sequences as identified by the VSEARCH algorithm (55). Taxonomic assignment followed using EzBioCloud reference (54). Mitochondrial, archaeal and eukaryotic sequences were removed. Operational taxonomic units (OTU) based analyses were performed on a random subset of 1360 reads per sample with a cutoff level of 0.03 or based on taxonomic assignment.

### Flow cytometry analysis

100 µl EDTA blood were stained with 1 µl Fixable Viability Dye and with antibodies for T-cell subpopulation differentiation (CD3, CD4, CD25, Foxp3) and intracellular cytokines, chemokines and transcription factors (RORγt, IFN-γ, IL-2, IL-8, IL-17A, IL-10 and TGF-β, **Supplementary Table 2**) using Foxp3/Transcription Factor Staining Buffer Set (Thermo Fisher Scientific, Waltham, USA), which includes a lysis buffer for red blood cell lysis, and 50 µl Brilliant Stain Buffer (BD Biosciences, Becton, Dickinson and Company, Franklin Lakes, USA). Stained cells were resuspended in 500 µl Flow cytometry staining buffer (Thermo Fisher Scientific, Waltham, USA) and analyzed via flow cytometry (BD LSR II, BD Biosciences, Becton, Dickinson and Company, Franklin Lakes, USA) within four days. Compensation controls were carried out using beads (Invitrogen, Thermo Fisher Scientific, Waltham, USA) for generating a compensation matrix. The gating strategy included gating for singlets, alive cells, T-lymphocytes, T helper cells (CD3+ CD4+), cytotoxic T cells (CD3+ CD4-) and regulatory T cells (CD3+ CD4+, Foxp3+ CD25+). Fluorescence minus one controls were used to determine gates for cytokines, chemokines and transcription factors.

### Statistical analysis

Microbiome data – Statistical analyses and data visualization were performed in R version 4.0.1 (r-project.org). Alpha diversity was compared using Shannons diversity index computed in psych package (56) and by calculating number of species in vegan package (57). Differences between groups were analyzed via Kruskal-Wallis-Test and pairwise Wilcoxon rank-sum test. To further assess differences between groups, indicator species were derived using Linear Discriminant Analysis Effect Size (LEfSe) (58) powered by Galaxy Project Platform (59). Score for differentiating discriminative features was set at 2.0. Beta diversity was analyzed applying principal coordinate analysis based on Bray-Curtis dissimilarities in labdsv package (60) and constrained correspondence analysis in vegan package (57). Differences between the groups were assessed via analysis of variance permutation testing in vegan package (57). Correlations between alpha diversity and antibiotic exposure degree were derived via Pearsons correlation coefficient in psych package (56).

Flow cytometry data – Relative expression as well as median fluorescence intensity (MdFI) of cytokines, chemokines and transcription factors were statistically analyzed with the Mann-Whitney-U-Test in R (version 4.3.2, packages *tidyverse, dplyr, ggpubr*). Statistical significance was defined as alpha-error < 5%. Because of alpha error accumulation, the significance level was adjusted for multiple testing using the Bonferroni correction.

Descriptive cohort data – Analysis was conducted in R (version 4.3.2) using Fishers exact test or Pearsons Chi-squared test with Yates continuity correction or Mann-Whitney-U-Test, statistical significance was defined as alpha-error < 5%. The influence of the count of penicillin doses on penicillin concentrations in cord blood was determined via linear regression, therefore the packages *tidyverse, sandwich, ggfortify* and *car* were applied.

## Results

### Study population

48 mothers and their infants were enrolled, 26 were allocated to the control group and 22 to the IAP exposed group. At birth, the two groups did not differ with respect to main perinatal parameters (**Table 1**, **Supplementary Table 3**). Follow up rate at one month and one year was 100% including a full set of samples.

**Table 1:** Main characteristics of participating infants did not differ between IAP-exposed and control neonates at birth.

	all	controls	IAP-exposed	p-value	test
**n**	48	26	22		
gestational age at birth [weeks], median (1.-3. quartile)	40.1 (39.4-41.0)	40.1 (39.5-41.1)	40.1 (39.2-40.6)	0.3728	§
birth weight [grams], median (1.-3. quartile)	3635 (3225-4100)	3795 (3285-4138)	3475 (3190-3822)	0.1993	§
small for gestational age (SGA), n (%)	5 (10.4)	2 (7.7)	3 (13.6)	0.6492	o
female, n (%)	31 (64.6)	16 (61.5)	15 (68.2)	0.8598	#
APGAR 1, median (1.-3. quartile)	9 (8-9)	9 (9-9)	9 (8-9)	0.1319	§
APGAR 5, median (1.-3. quartile)	10 (9-10)	10 (9-10)	10 (9-10)	0.715	§
APGAR 10, median (1.-3. quartile)	10 (10-10)	10 (10-10)	10 (10-10)	0.5283	§

Mann-Whitney-U-Test (§), Fishers Exact Test (o), Pearsons Chi-squared test with Yates continuity correction (#).

IAP exposed vs. control infants showed no differences in feeding type, antibiotic use or probiotic supplementation and hospitalization at one month and one year. Secondary outcomes such as weight, z-scores for weight and body mass index (BMI), occurrence of infections and other health-related outcomes were not different among IAP exposed and control infants during the first year of age (**Supplementary Table 3**).

### Age dependent change of microbiota

**Figure 1:**
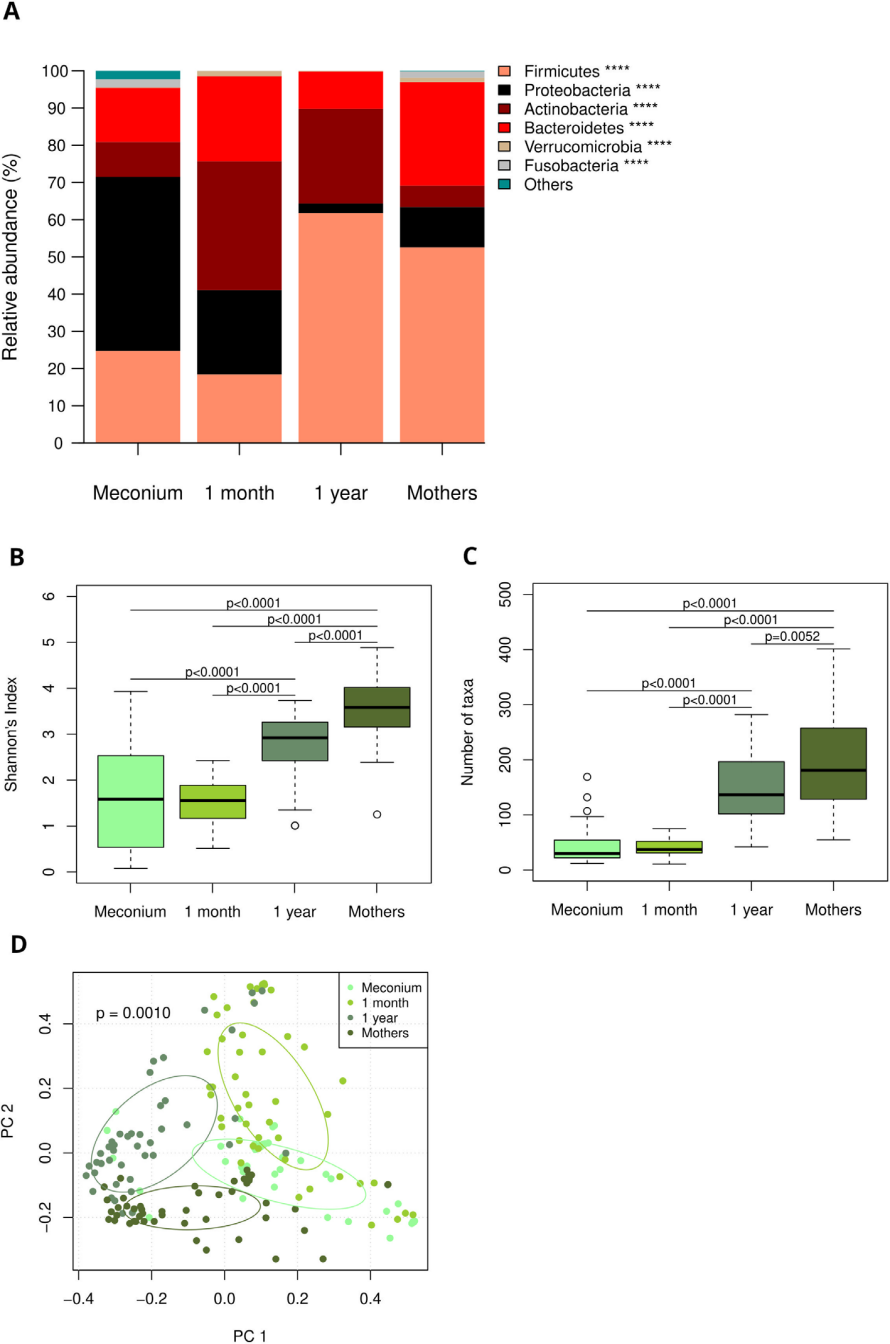
**(A)** Age dependent and age specific microbiome composition at birth, one month and one year of age and in maternal swabs, depicted as relative abundances of most abundant phyla. Taxa are depicted in the legend with their respective significance value (Kruskal-Wallis test: ****p<0.0001) **(B)** Shannons diversity index at birth, one month and one year of age as well as in maternal swabs (Pairwise Wilcoxon rank-sum test). **(C)** Number of detected species at birth, one month and one year of age as well as in maternal swabs (Pairwise Wilcoxon rank-sum test). **(D)** Principal component analysis reveals distinct clusters for each age group and maternal samples.

Analysis of V3/V4 hypervariable region of the 16S rRNA gene revealed age dependent and age specific microbiome compositions at birth, one month of age and one year of age. **Figure 1** illustrates data of the entire cohort, including both groups and all feeding types. In meconium samples, *Proteobacteria* dominated the infant gut, whereas at the age of one month a pronounced abundance of *Actinobacteria* (particularly *Bifidobacterium (B.) longum*) was notable. At the age of one year, the infant gut microbiome was dominated by *Firmicutes* (**Figure 1A**, species level data in **Supplementary Figure 1**). Both microbiome diversity and number of species increased with age (**Figures 1B, C**). Principal component analysis revealed distinct clusters for each age group as well as maternal swabs (**Figure 1D**).

### IAP with penicillin has limited effects on meconium microbiome

The microbiome of meconium samples showed minimal alterations with regard to IAP. Linear discriminant analysis Effect Size (LEfSe) revealed that *Lactobacillales* were a signature taxon in control infants. *Negativicutes* (species *Veillonella dispar* and *Dialister invisus*) and *Stigonematales* as well as the species *Enterococcus gilvus, Bacteroides uniformis* and *B. bifidum* and the genera *Sporobacter*, *Citrobacter* and *Kluyvera* were associated with exposure to IAP (**Supplementary Figure 2C**). Global measures of microbiome diversity and relative abundances did not differ significantly between groups (**Supplementary Figures 2 A, B, D, E**).

### IAP leads to reduced expansion of *Bifidobacterium longum* at one month of age

**Figure 2:**
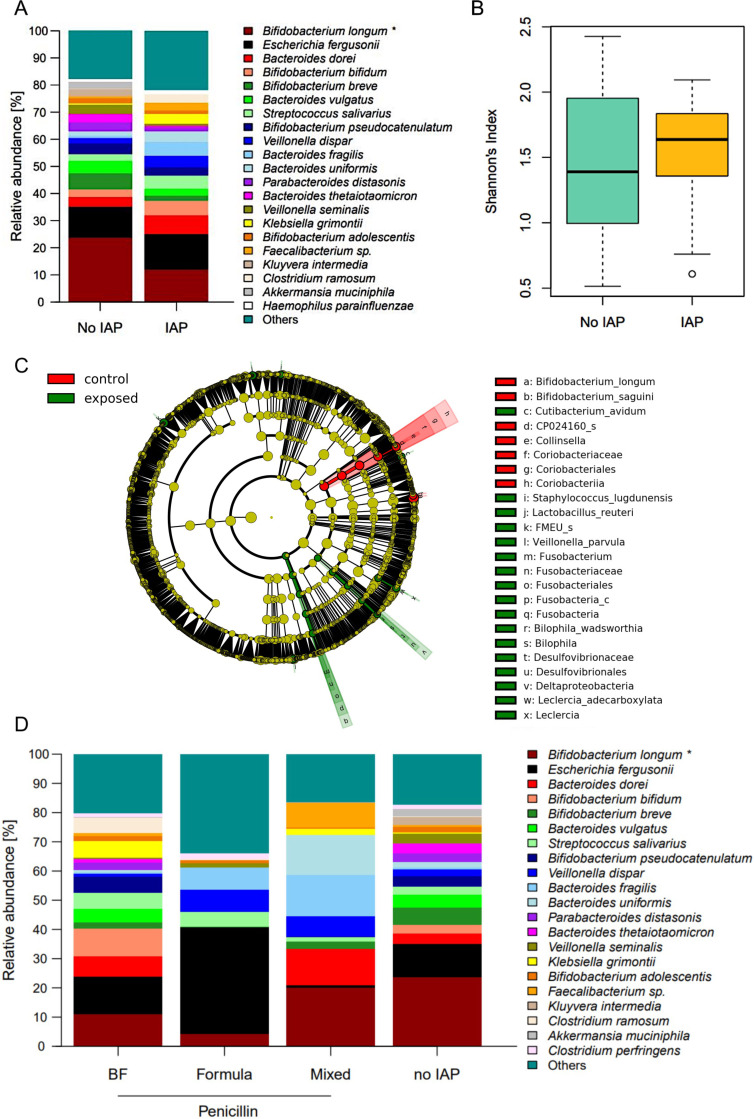
**(A)** Relative abundances of the most abundant species in fecal samples at one month. Significantly higher abundance of B. longum in control infants compared to IAP exposed infants. Taxa are depicted in the legend with their respective significance value (Mann-Whitney-U-Test: *p = 0.02). **(B)** Shannons diversity index of fecal mibrobiome samples at one month did not differ between exposed and control infants. **(C)** Influence of nutrition on relative abundances in fecal microbiome at one mont. Taxa are depicted in the legend with their respective significance value (Kruskal-Wallis test: *p = 0.042). BF = exclusively breast fed infants, formula = exclusively formula fed infants, mixed = infants fed with breast milk and formula. **(D)** .

In contrast to meconium samples, a stronger effect of IAP was detected in stool samples at the age of one month. The relative abundance of *B. longum* was significantly higher in control infants compared to exposed infants (23.8% vs. 12.0%, p = 0.02, **Figure 2A**). Other bifidobacterial species, such as B. breve and B. adolescentis, were nonsignificantly reduced in IAP-exposed infants at one month (**Figure 2A**). There was a significant impact of nutrition on the relative abundance of *B. longum* (breastfed neonates 10.9%, formula-fed neonates 4.2%, mixed [i.e. feeding of breast milk and formula] 20.1%, Kruskal-Wallis-Test p = 0.042, **Figure 2D**), whereas testing for specific feeding habits revealed no significant impact, possibly due to correction for multiple testing. In line with the relative abundances, *B. longum* was identified as a signature taxon for control infants at one month. Additionally, the species *B. sanguini*, the genus *Collinsella* and family, order and class *Coriobacteriaceae, Coriobacteriales* and *Coriobacteriia* were detected as signature taxa in control infants (**Figure 2C**). Indicator species for IAP exposure at one month of age were *Deltaproteobacteria* (with species *Bilophila wadsworthia*), *Fusobacteria* (genus *Fusobacterium*), *Leclercia* (*Leclercia adecarboxylata*) as well as the species *Cutibacterium avidum*, *Staphylococcus lugudunensis*, *Lactobacillus reuteri* and *Veillonella parvula* (**Figure 2C**). Global measures of microbial diversity were not affected by IAP (**Figure 2B**, **Supplementary Figures 3A, B**).

### Effects of IAP are sustained through the first year of age

**Figure 3:**
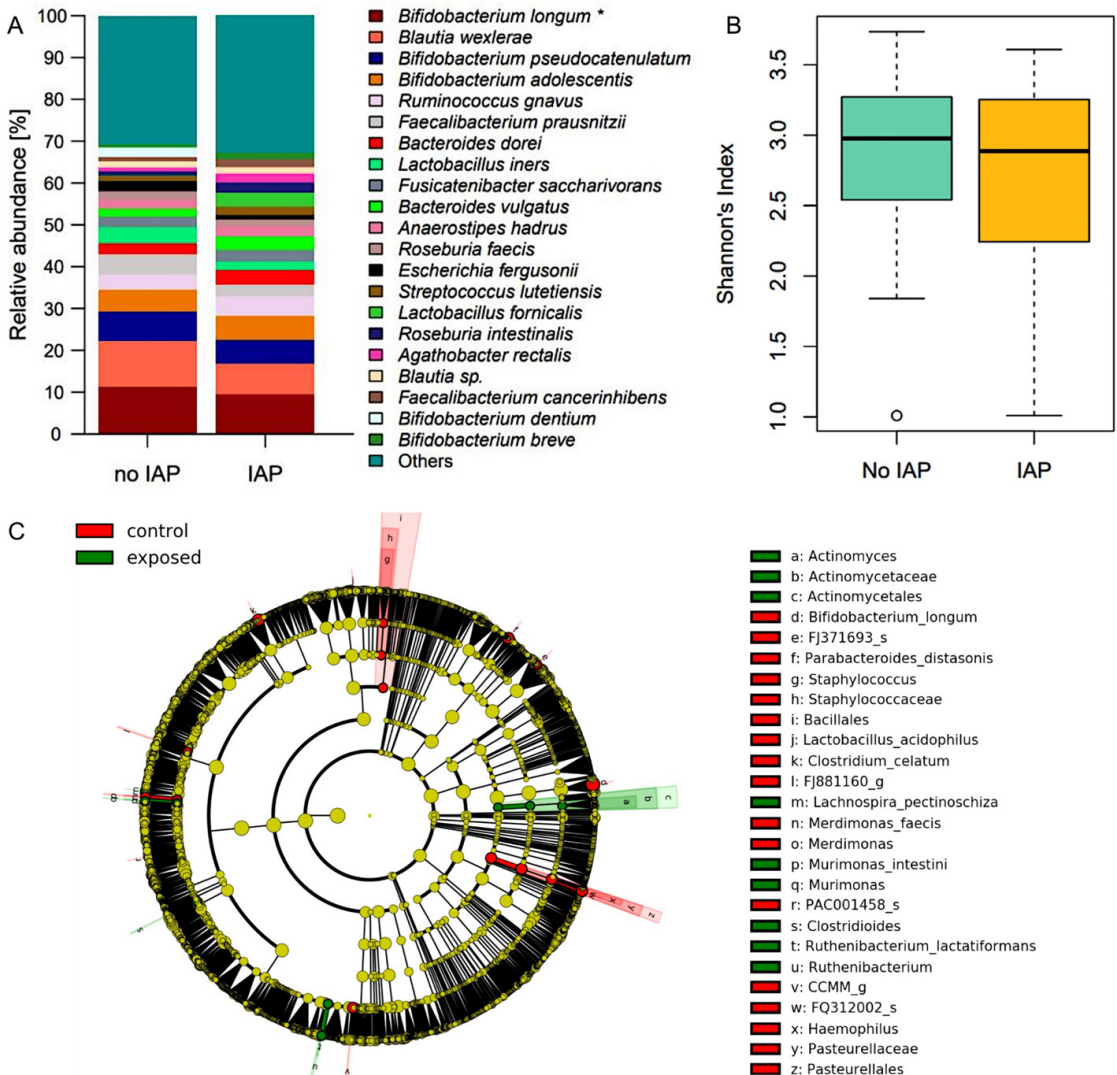
**(A)** Relative abundances of the most abundant species in fecal samples at one year. Significantly higher abundance of B. longum in control infants. Taxa are depicted in the legend with their respective significance value (Mann-Whitney-U-Test: *p = 0.044). **(B)** Shannons diversity index of fecal mibrobiome samples at one year did not differ between exposed and control infants. **(C)** LEfSe analysis of fecal microbiome at one year identified signature taxa in IAP exposed and control infants.

As in one month samples, the relative abundance of *B. longum* was persistently reduced in IAP exposed infants at the age of one year (9.5% vs. 11.4%, p = 0.044, **Figure 3A**). Consistent with the results at one month, indicator species analysis revealed *B. longum* as a signature taxon for control infants. Moreover, Bacillales, *Pasteurellales*, *Merdimonas* as well as the species *Parabacteroides distasonis* and *Lactobacillus acidophilus* and *Clostridium celatum* were identified as signature taxa for control infants.

On the contrary, *Actinomycetales*, *Clostridioides*, *Murimonas* and the species *Lachnospira pectinoschiza* and *Ruthenibacterium lactatiformans* were associated with IAP in these samples (**Figure 3C**). Global measures of microbiome diversity did not differ between groups (**Figure 3B**, **Supplementary Figures 3C, D**).

### IAP exposed infants display a proinflammatory T helper cell repertoire

**Figure 4:**
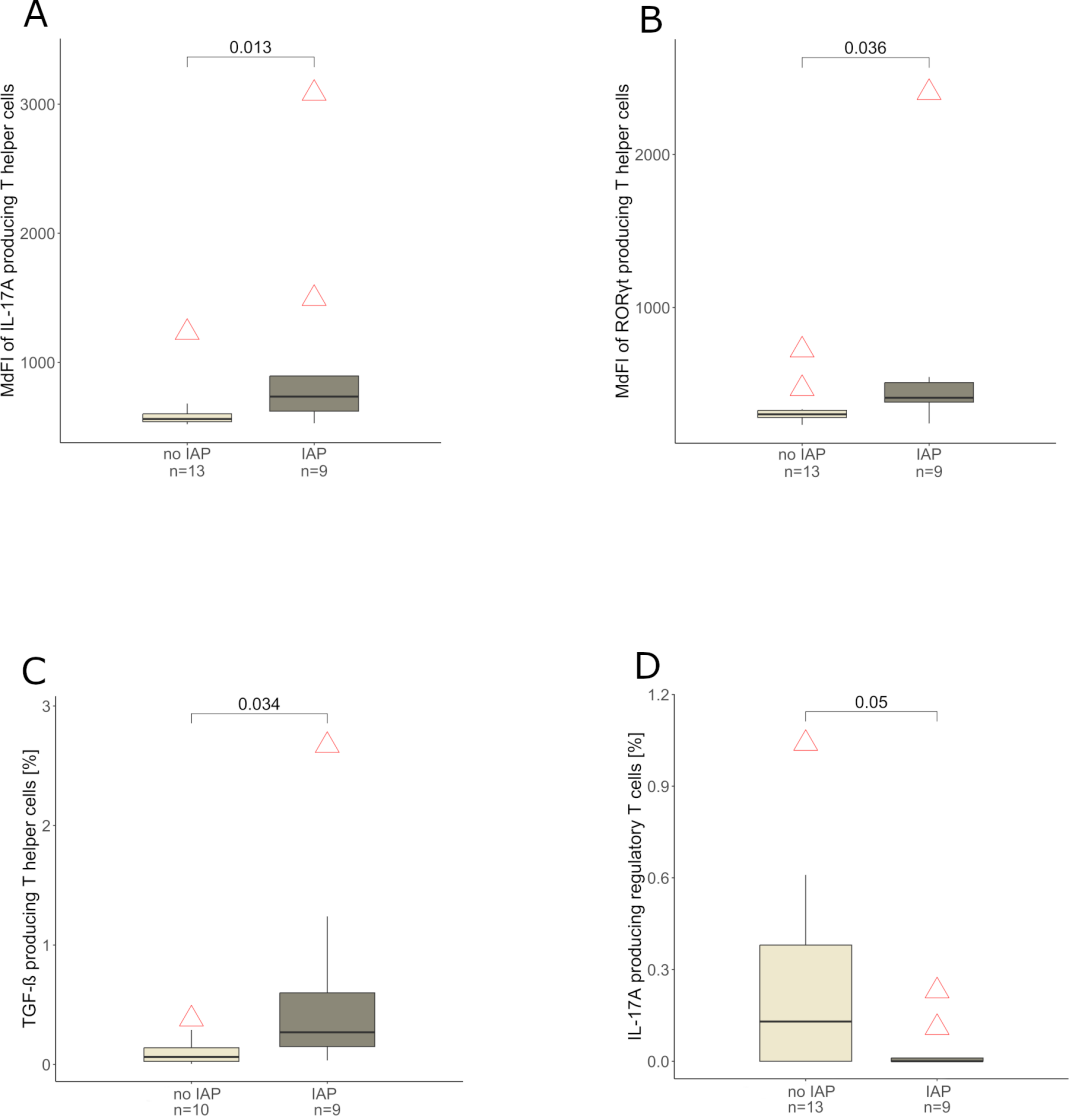
**(A, B)** Median Fluorescence Intensity (MdFI) of T helper cells producing IL-17A and RORγt derived from peripheral blood of infants without and with IAP respectively, light grey: no IAP, dark grey: IAP exposure. **(C)** T helper cell frequencies [%] producing TGF-β derived from peripheral blood of infants without and with IAP respectively, light grey: no IAP, dark grey: IAP exposure. **(D)** Regulatory T cell frequencies [%] producing IL-17A derived from peripheral blood of without and with IAP respectively, light grey: no IAP, dark grey: IAP exposure.

**Figure 5:**
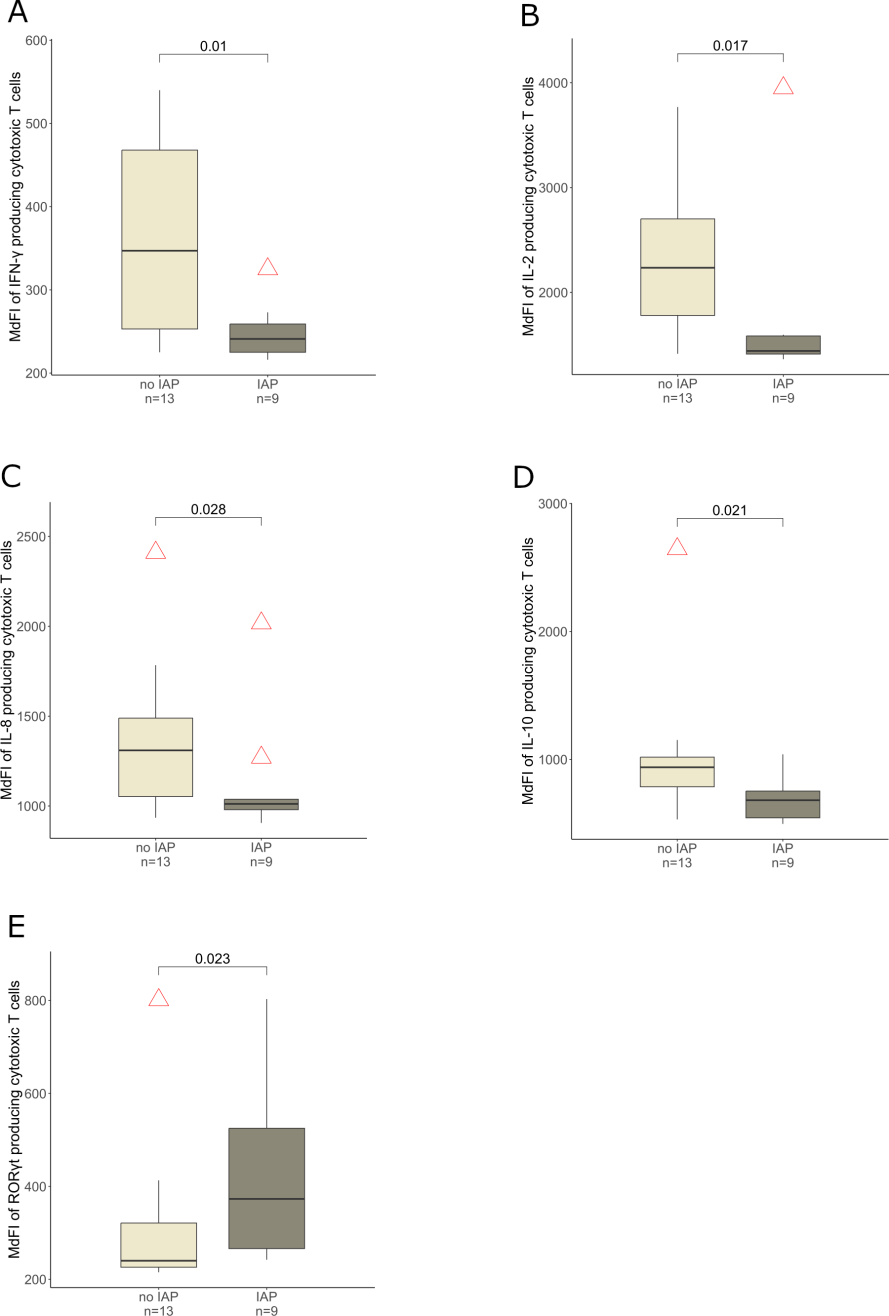
MdFI of cytotoxic T cells producing **(A)** IFN-γ, **(B)** IL-2, **(C)** IL-8, **(D)** IL-10 and **(E)** RORγt derived from peripheral blood of infants without and with IAP respectively, light grey: no IAP, dark grey: IAP exposure.

The subset of the whole cohort which provided blood samples (22 of 48 infants, n = 9 IAP exposed and n = 13 control infants) showed no significant differences among IAP exposed and control infants concerning important characteristics such as gestational age, occurrence of early onset disease, birth weight and sex (data not shown). IAP was associated with a higher expression of IL-17A (p = 0.013), RORγt (p = 0.036) and TGF-β (p = 0.034) in T helper cells (**Figures 4A–C**) in peripheral blood during the first days after birth. Interestingly, regulatory T cells expressed less IL-17A in IAP exposed infants compared to control infants (p = 0.05, **Figure 4D**). Cytotoxic T cells (defined as CD3+ CD4- lymphocytes) expressed less IFN-γ (p = 0.01), IL-2 (p = 0.017), IL-8 (p = 0.028) and IL-10 (p = 0.021) in IAP exposed infants, whereas a higher expression of RORγt (p = 0.023) was observed (**Figures 5A–E**). Flow cytometry data did not remain significant after Bonferroni-correction for multiple testing.

### Repetitive penicillin doses have no cumulative effect on microbiome diversity

The median penicillin concentration of 12 available cord blood samples was 6.3 µg/ml. We observed no cumulation of penicillin-concentrations in cord blood with increasing penicillin administrations (**Supplementary Figure 4B**). In a subgroup analysis at one month of age, the Shannons diversity index did not correlate with the number of penicillin doses (**Supplementary Figure 4A**).

## Discussion

GBS is a leading cause of EOD in term newborns (1) (2). Its vertical transmission during birth can be prevented with IAP. In order to explore effects of an IAP on the fecal infant microbiome in the first year of age as well as the T cell phenotype in the first days after birth, we conducted a prospective cohort study of 48 mothers and their term infants, among them 22 with IAP (here defined as penicillin G for maternal GBS-colonization) and 26 without any *intrapartum* antibiotics (control group).

We found that IAP significantly reduced the relative abundance of *B. longum* in the infant gut at one month and one year of age. In infant blood, a predominantly proinflammatory T-helper-phenotype in penicillin-exposed neonates was observed with a higher production of IL-17A, RORγt and TGF-β.

In meconium, multiple facultative anaerobic colonizers were identified, such as *Escherichia*, *Enterococcus* and *Staphylococcus*, which are typical pioneer colonizers of the infant gut (61). *Lactobacillus* and *Prevotella* were also present in meconium, most likely as a result of vaginal birth (62). *B. longum* occurred with a relative abundance of 0.4% and 2.3% in control vs. exposed infants, respectively.

Strict anaerobic colonizers such as *Bifidobacterium* and *Bacteroides* became more abundant at one month postpartum, consistent with previous findings (35). *B. longum* abundance was significantly reduced in IAP exposed infants.

At one year, mainly *Bifidobacterium* and *Bacteroides* dominated the infant gut. As described by Bäckhed et al., signature taxa at 12 months, such as *Bacteroides, Anaerostipes*, and *Roseburia* were also present in our cohort (61). Notably, one third of the cohort was still breastfed at one year, maintaining taxa like *Bifidobacterium* and *Lactobacillus* (61) (63). The relative abundance of *B. longum* remained significantly lower in IAP exposed infants compared to control infants at one year.

*Bifidobacteria* dominate the gut microbiome of breastfed infants during the first year and *B. longum* declines in abundance throughout life (64). It is known to mediate multiple beneficial effects in early infancy: *B. longum subspecies infantis* is the champion metabolizer of human milk oligosaccharides (65), converts these into acetate and provides hereby additional energy for the infant (19) (65) (66). *B. longum* protects against enteropathogenic infections by producing acetate (18) and by lowering fecal pH (20). *Bifidobacteriaceae* promote intestinal tolerance by inducing regulatory T cells and anti-inflammatory cytokines like IL-10 and IL-27 (67), which could be particularly important in the immune regulation of microbiota-induced immune responses during colonization of the newborn. Bifidobacteria-derived indol-3-lactic acid (68) suppresses T_H_17 responses via Galectin-1 (67), providing a possible functional link between the proinflammatory immune phenotype of T helper cells (IL-17A, RORγt and TGF-β) and diminished *Bifidobacterium longum* observed in IAP exposed infants in our study. Galectin-1, a β-galactoside binding lectin (69), modulates T-cell functions by reducing cell adhesion to extracellular matrix (70) and endothelial cells (71) and through an altered cytokine production towards antiinflammation (70) (72).

Reduced bifidobacterial counts are a feature of dysbiosis (73) and linked to intestinal inflammation in celiac disease (74) (75), colic (76), obesity (77), and autoimmunity (28). Reduced bifidobacterial abundances could also compromise vaccine responses as these were positively associated with *Bifidobacterium* and *Bifidobacterium longum subspecies infantis* in a Bangladeshi cohort at 15 weeks of age (78). Thus, reduced *B. longum* abundances might have relevant short term as well as long term consequences for the infant health and development.

The effects of IAP on the infant microbiome in vaginally delivered term infants have been investigated extensively, with studies focusing on the first weeks after birth and some following up to one year (29–44). A reduction in *Bifidobacterium* following IAP has been observed between one and twelve weeks (30) (31) (33) (34), (35), (37), (39), (42). In the largest cohort of vaginally delivered, IAP-exposed (n=375) and not exposed infants (n=876) studied to date, a reduction of *Bifidobacterium* was described in partially or fully breastfed infants at the age of three months in both, 16S rRNA-Analysis and qPCR (42). At family-level, a reduction of *Bifidobacteriaceae* after IAP exposure was observed between the age of two days and four weeks in vaginally delivered terms (36) (38), including one study showing remarkably increased *Bifidobacteriaceae* at the age of 12 month (36).

Furthermore, recent research regarding microbial deviations following IAP in vaginally delivered term infants consistently reports a reduction in *Bacteroides* (between the second day after birth and 12 months) (29) (33) (40) (44). Also, elevation of *Clostridium* in the infant feces (between one and 12 month of age) (29) (40) (41), was observed, whereas one study detected lower *Clostridium* at 12 months (33).

We identified several signature taxa for IAP exposed and non-exposed infants during the first year of age. *B. longum*, consistently identified in non-exposed infants at one month and one year, is associated with numerous beneficial effects, as described above. In meconium, *Lactobacillales*, typical early colonizers in vaginally born infants, are a signature taxon for non-exposed infants, indicating a reduced transmission of *Lactobacillales* during parturition from the maternal vaginal microbiome in IAP-exposed newborns (62). *Lactobacillales* typically reside in the intestines of vaginally delivered, breastfed infants (62) (63), exerting beneficial effects such as enhancing tight junction integrity and reducing the adhesion of enteropathogenic bacteria (23). At one month, *Coriobacteriaceae* was a signature taxon in non-exposed infants fecal samples, which may protect against intestinal inflammation (79). In contrast, *Fusobacterium* was a signature taxon in IAP exposed infants at one month. This is intriguing, as *Fusobacterium* promotes a proinflammatory microenvironment (80) and *Fusobacteriaceae* can be indicative of disease status in treatment-naïve Crohns disease in pediatric patients (81).

The proinflammatory T helper cell phenotype (TGF-β, RORγt, IL-17A, **Figure 4A-C**) among IAP exposed neonates in our small sample study generates the hypothesis that T_H_17-cell responses might be pronounced in IAP-exposed neonates. T_H_17 cells are involved in acute inflammatory reactions (82), strengthen the mucosal barrier function (83) and ward off pathogens (84). Depending on IL-23, they can induce autoimmunity (85) and adopt a pathological phenotype (86). T_H_17-cells are involved in the pathogenesis of psoriasis (87) and are frequently present in patients with multiple sclerosis (88). In mice, delayed microbial maturation increased T_H_17-frequency and led to IL-17A dependent aggravated allergic asthma, even with allergen exposure at the time of normalized microbial diversity (89). Consistently, a higher RORγt expression in CD3+ CD4- cells among IAP exposed neonates (**Figure 5E**) could indicate an increased T_c_17 cell response, defined as CD8 positive T cells producing IL-17 (90), which play an important role in inflammatory diseases (91) (88) (92).

RORγt positive regulatory T cells are induced in mice by microbiota and their metabolites (93) (94), and are also found in human colonic biopsies (94). Human RORγt positive regulatory T cells can produce IL-17 under proinflammatory conditions *in vitro*, temporarily losing their suppressive functions (95). Al Nabhani et al. describe a “weaning reaction” in mice, an intestinal immune response during a defined time window, where disturbances like antibiotics can cause lifelong inflammatory imprinting. During this reaction, microbiota and their metabolites induce RORγt positive T_regs_ (96). We observed a reduction in IL-17A-producing T_regs_ in IAP exposed infants, possibly due to an impaired microbiota-dependent induction of RORγt positive T_regs_ *. In vitro*, TGF-β inhibits IL-17 production in Foxp3+ cells (95), possibly explaining the reduction in IL-17A-producing T_regs_ (**Figure 4D**), whereas CD4 positive cells exhibited a higher TGF-β production (**Figure 4C**) in IAP exposed infants in our study.

We observed a reduced activity of cytotoxic T cells in IAP exposed infants e.g. in the production of IFN-γ and IL-2 (**Figure 5A, B**). Although interferon responses are important to combat viral infections, our T cell findings did not translate into differences in the occurrence of infections during the first year of age.

In an available subgroup of 12 cord blood samples, 10/12 of penicillin-concentrations were above the minimal inhibitory concentration for GBS-Meningitis of 0.125 µg/ml (97), indicating efficacious concentrations in mothers and infants. A median penicillin concentration in cord blood of 6.3 µg/ml was reached, penicillin concentrations measured in this study are in line with the published cord blood concentrations of penicillin as IAP (98) (99).

A major strength of this study lies in the prospective study design and a balanced composition of the cohort consisting of vaginally born term-infants at least partially breastfed over 80% at the age of one month and approximately one third of the cohort at the age of one year. No family was lost to follow-up and even over 80% of the meconium samples provided by IAP exposed and control infants were included in the final microbiome analysis. There are only a few studies investigating effects of IAP on the infant microbiome during the entire first year of age (29) (32) (33) (36), (44), none of them integrating immune responses of the infant.

Limitations, on the other hand, lie in a rather small sample size and possible confounding factors such as postpartum applied antibiotics (13% at the age of one month, 21% at the age of one year, there were no significant differences between IAP exposed and control infants, **Supplementary Table 3**).

We used partial 16S sequencing, which is known to have limited precision in species level assignment in analyzing the microbiota (100). More precise and accurate classification could have been achieved using full length 16S sequencing (101). While we are aware of that species level assignments are to be taken with caution, we believe species level assignment to be inevitable in neonatal research and we have outlined usability of our approach in recent studies (e.g (102–104)) and believe this to be an adequate strategy in a setting with a focus on neonatal microbial communities which provide less diversity and thus less complexity. Examination of T cell panels during the first days after birth was performed only in a subset of infants (n = 22) and might therefore be underpowered. A longer follow-up period of a larger cohort during early childhood could account for dysbiosis-mediated diseases as a possible result of inflammatory imprinting of the immune system, such as obesity and diseases with intestinal inflammation such as colic, celiac disease or Crohns disease.

Increasing the abundance of *B. longum subsp*. *infantis* by probiotic supplementation might be an approach to prevent or alleviate IAP induced dysbiosis and potential harmful long-term consequences in infants at risk. Twenty-one days of *B. longum subsp*. *infantis* strain EVC001-supplementation in breastfed newborns led to a sustained increase in bifidobacterial counts and a reduction of inflammatory surrogate parameters (less fecal IL-17A, higher anti-inflammatory indol-3-lactic acid and less fecal calprotectin) (20) (67) (105).

Following IAP in GBS-colonized mothers, the relative abundance of *B. longum* was significantly reduced at the age of one month and this reduction persisted up to one year. IAP was furthermore associated with a proinflammatory T-helper-phenotype during the first days of age. Our findings suggest that intrapartum antibiotic prophylaxis can have a sustained impact on the infant gut microbiome and early immune development. These observations may help to better understand the potential consequences of early-life interventions and their role in shaping microbiota and immune function. Further research with adequate power and high-quality investigation methods in the field of IAP-induced dysbiosis and health-related outcomes with a longer follow-up than one year is needed.

## Data Availability

The raw sequencing data are freely available online at the European Nucleotide Archive at https://www.ebi.ac.uk/ena/browser/home under accession number PRJEB80487.

## Supplementary material

Supplementary Figure 1 Relative abundances of the most abundant species in meconium samples as well as samples at one month and one year of age and maternal swabs.

Supplementary Figure 2
**(A)** Relative abundances of the most abundant species in meconium samples showed little differences between IAP exposed and control newborns. **(B)** Shannons diversity index of meconium samples did not differ between exposed and control infants. **(C)** LEfSe analysis of fecal microbiome in meconium samples identified signature taxa in IAP exposed and control infants. **(D)** Beta diversity showed no significant differences between IAP exposed and control infants in meconium samples **(E)** Constrained correspondence analysis indicated that IAP exposure explained 2.3% of compositional variance.

Supplementary Figure 3
**(A)** Beta diversity showed no significant differences in fecal samples at one month between IAP exposed and control infants. **(B)** Constraining for IAP exposure displayed 2.23% of compositional variance explained by the selected variable at the age of one month. **(C)** Beta diversity showed no significant differences in fecal samples at one year between IAP exposed and control infants. **(D)** Constrained correspondence analysis showed 1.7% of variance explained by IAP exposure at the age of one year.

Supplementary Figure 4
**(A)** Shannons diversity index and number of penicillin applications at one month of age showed no significant Pearsons product-moment correlation**. (B)** Penicillin concentrations in cord blood and number of penicillin applications show no significant correlation in linear regression.

Supplementary Figure 5 Gating strategy. After singlet discrimination, gating of live cells and CD3+ lymphocytes (light green), T helper cells (CD4+) and cytotoxic T cells (here defined as CD3+CD4-) were discriminated. Within each T cell subtype different antigens were detected (exemplary shown here with T helper cells).
