# Supplementary figures and images for: Persistent reduction of Bifidobacterium longum in the infant gut microbiome in the first year of age following intrapartum penicillin prophylaxis for maternal GBS colonization
Source: Front Immunol. 2025 May 15;16:1540979. doi: 10.3389/fimmu.2025.1540979 (PMC12119681; doi:10.3389/fimmu.2025.1540979)

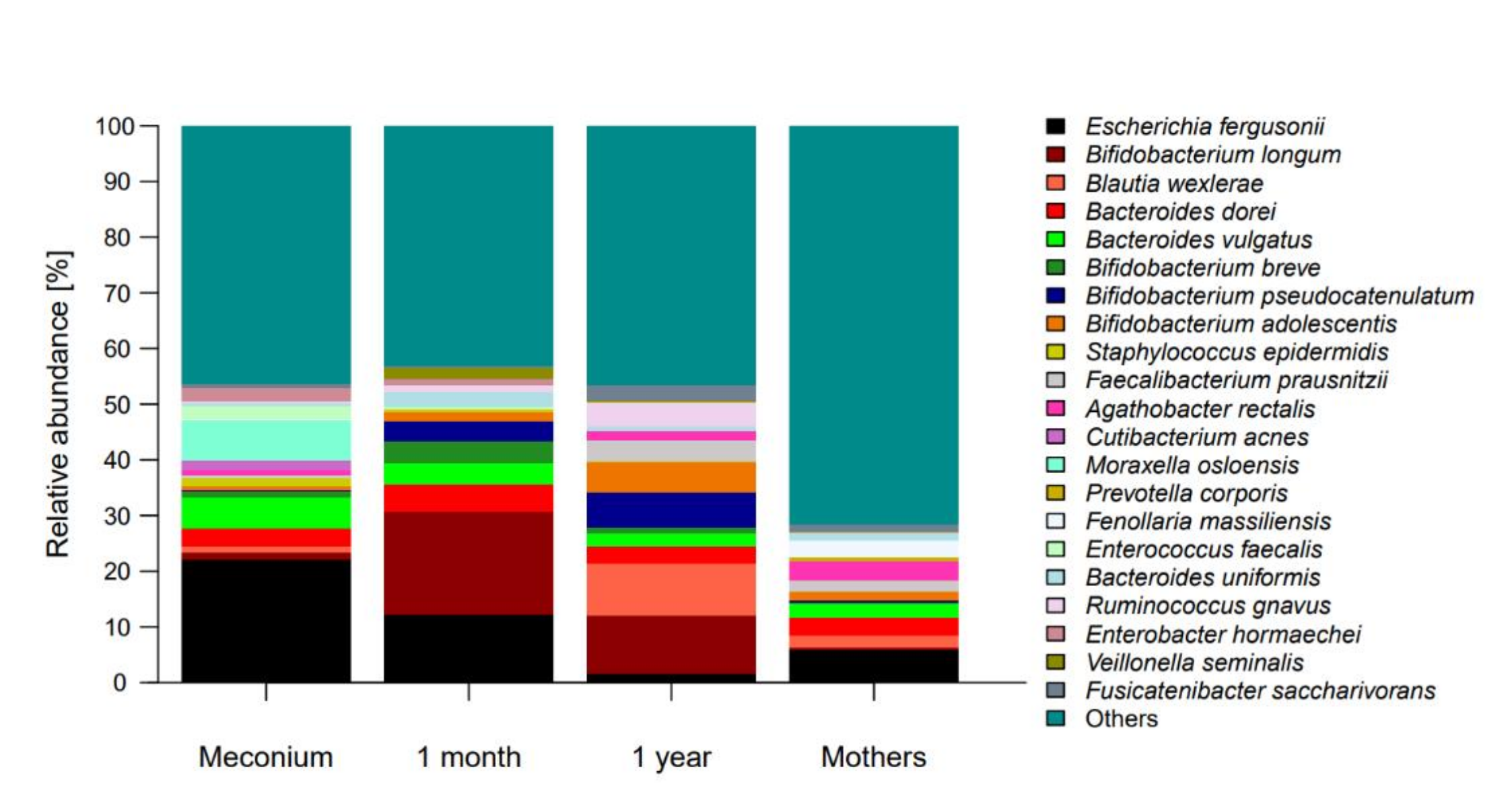

Supplement: Supplementary Figure 1 — Relative abundances of the most abundant species in meconium samples as well as samples at one month and one year of age and maternal swabs. [file Image1.tif]

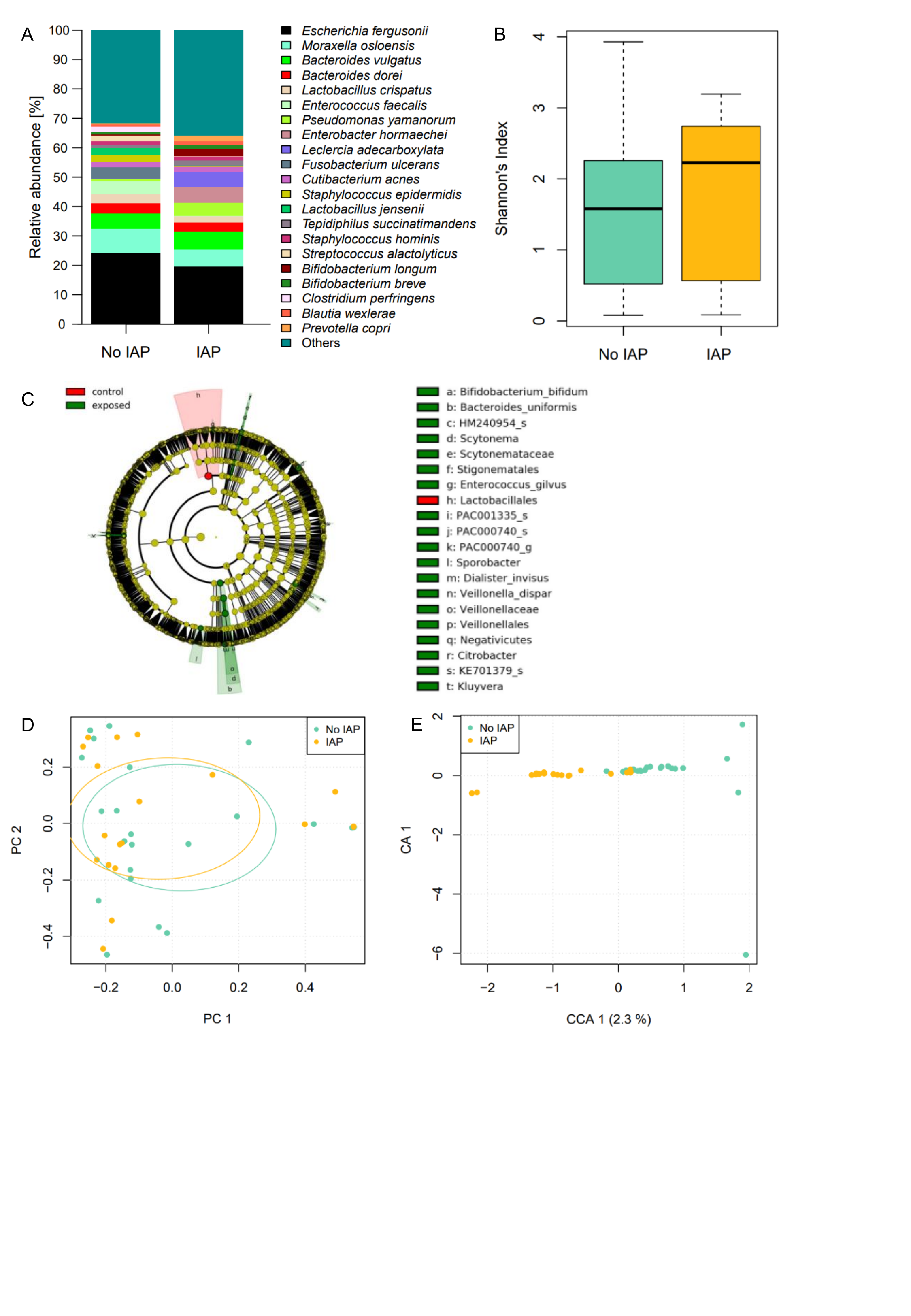

Supplement: Supplementary Figure 2 — (A) Relative abundances of the most abundant species in meconium samples showed little differences between IAP exposed and control newborns. (B) Shannons diversity index of meconium samples did not differ between exposed and control infants. (C) LEfSe analysis of fecal microbiome in meconium samples identified signature taxa in IAP exposed and control infants. (D) Beta diversity showed no significant differences between IAP exposed and control infants in meconium samples (E) Constrained correspondence analysis indicated that IAP exposure explained 2.3% of compositional variance. [file Image2.tif]

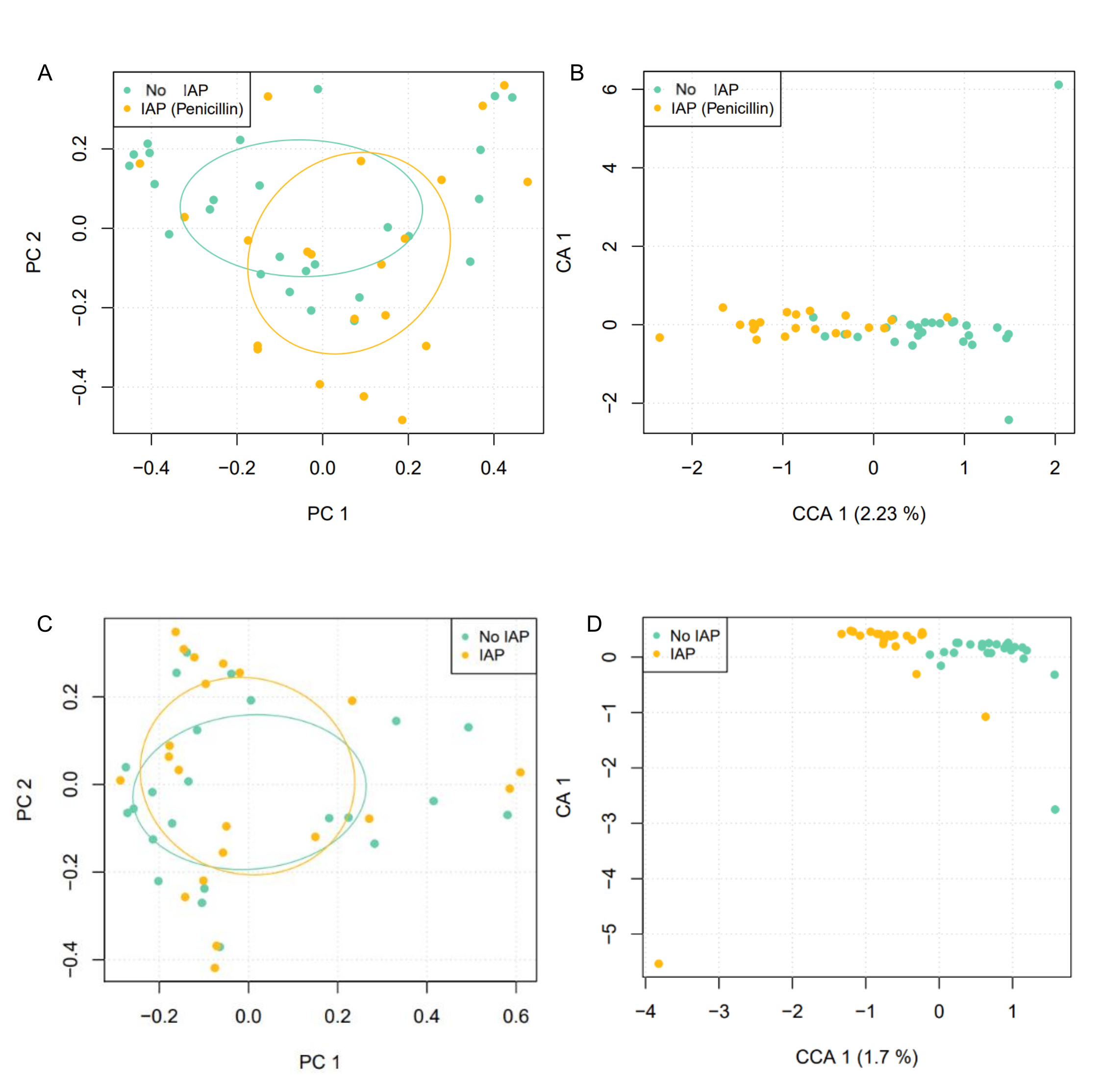

Supplement: Supplementary Figure 3 — (A) Beta diversity showed no significant differences in fecal samples at one month between IAP exposed and control infants. (B) Constraining for IAP exposure displayed 2.23% of compositional variance explained by the selected variable at the age of one month. (C) Beta diversity showed no significant differences in fecal samples at one year between IAP exposed and control infants. (D) Constrained correspondence analysis showed 1.7% of variance explained by IAP exposure at the age of one year. [file Image3.tif]

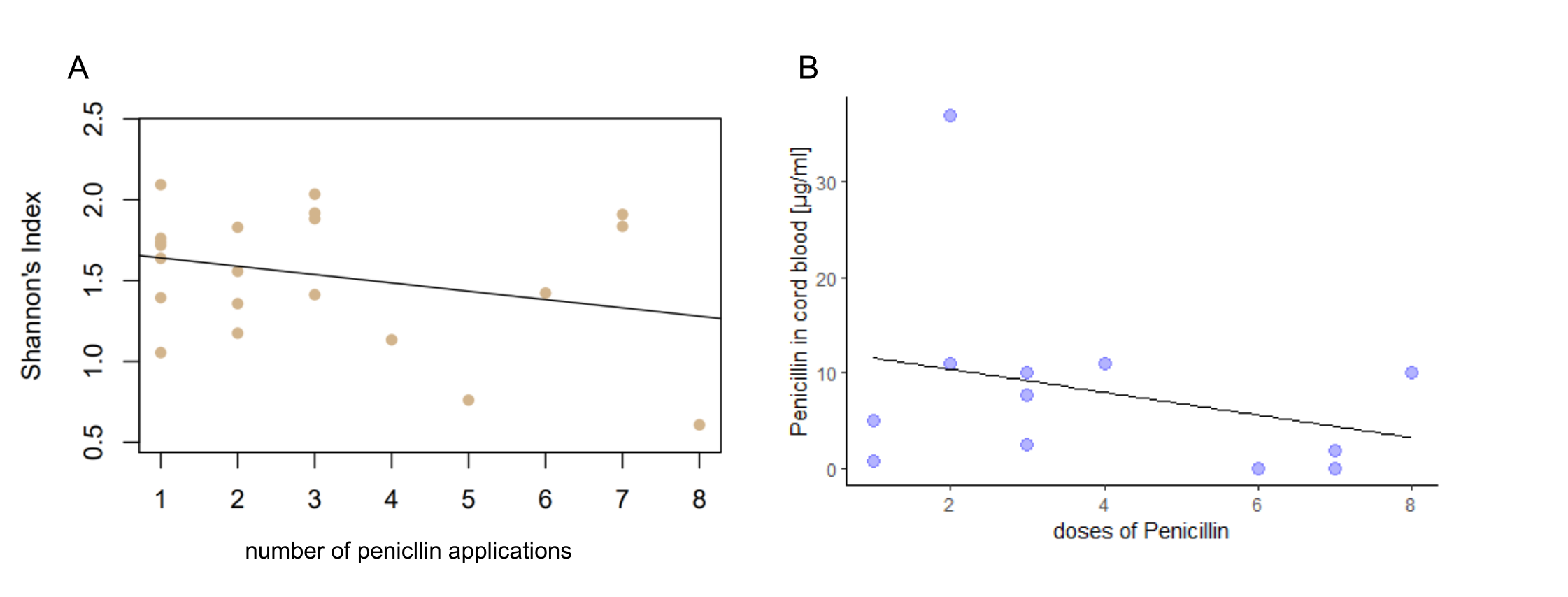

Supplement: Supplementary Figure 4 — (A) Shannons diversity index and number of penicillin applications at one month of age showed no significant Pearsons product-moment correlation. (B) Penicillin concentrations in cord blood and number of penicillin applications show no significant correlation in linear regression. [file Image4.tif]

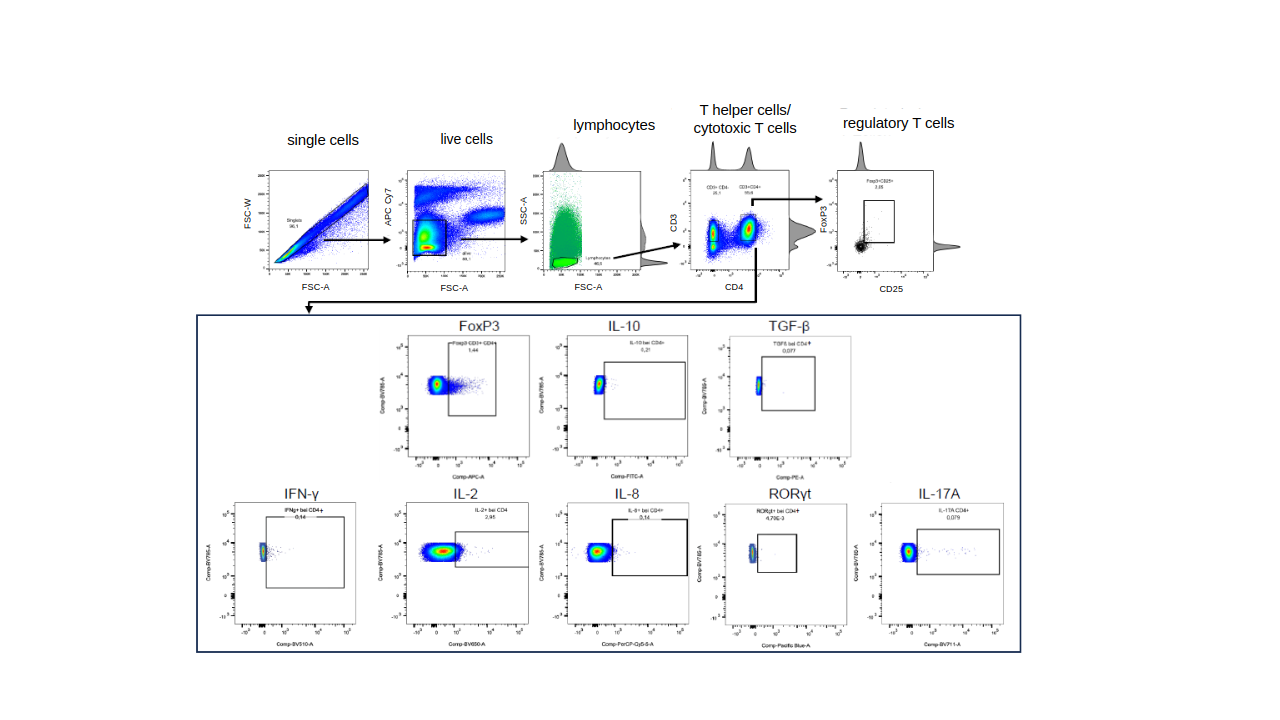

Supplement: Supplementary Figure 5 — Gating strategy. After singlet discrimination, gating of live cells and CD3+ lymphocytes (light green), T helper cells (CD4+) and cytotoxic T cells (here defined as CD3+CD4-) were discriminated. Within each T cell subtype different antigens were detected (exemplary shown here with T helper cells). [file Image5.tif]
