# Supplementary material for: Persistent reduction of Bifidobacterium longum in the infant gut microbiome in the first year of age following intrapartum penicillin prophylaxis for maternal GBS colonization
Source: Front Immunol. 2025 May 15;16:1540979. doi: 10.3389/fimmu.2025.1540979 (PMC12119681; doi:10.3389/fimmu.2025.1540979)
Supplement: Supplementary file 6 [file Table1.docx]

**Supplementary Table 1** List of used primers for 16S rRNA analysis of fecal samples

The table shows the primers for amplification of the V3/V4 hypervariable region of the16S rRNA gene used for the microbiome analysis of stool samples in the first year of age and maternal rectal swabs.

| Oligo Name | Sequence 5'-3' | P5/P7 | Index_i5 | Index_i7 |
| --- | --- | --- | --- | --- |
| hV3F_MID1 | AATGATACGGCGACCACCGAGATCTACAC ATCGTACG ACACTCTTTCCCTACACGACGCTCTTCCGATCT CCTACGGGAGGCAGCAG | i5 | ATCGTACG |  |
| hV3F_MID2 | AATGATACGGCGACCACCGAGATCTACAC ACTATCTG ACACTCTTTCCCTACACGACGCTCTTCCGATCT T CCTACGGGAGGCAGCAG | i5 | ACTATCTG |  |
| hV3F_MID3 | AATGATACGGCGACCACCGAGATCTACAC TAGCGAGT ACACTCTTTCCCTACACGACGCTCTTCCGATCT GT CCTACGGGAGGCAGCAG | i5 | TAGCGAGT |  |
| hV3F_MID4 | AATGATACGGCGACCACCGAGATCTACAC CTGCGTGT ACACTCTTTCCCTACACGACGCTCTTCCGATCT CGA CCTACGGGAGGCAGCAG | i5 | CTGCGTGT |  |
| hV3F_MID5 | AATGATACGGCGACCACCGAGATCTACAC TCATCGAG ACACTCTTTCCCTACACGACGCTCTTCCGATCT ATGA CCTACGGGAGGCAGCAG | i5 | TCATCGAG |  |
| hV3F_MID6 | AATGATACGGCGACCACCGAGATCTACAC CGTGAGTG ACACTCTTTCCCTACACGACGCTCTTCCGATCT TGCGA CCTACGGGAGGCAGCAG | i5 | CGTGAGTG |  |
| hV3F_MID7 | AATGATACGGCGACCACCGAGATCTACAC GGATATCT ACACTCTTTCCCTACACGACGCTCTTCCGATCT GAGTGG CCTACGGGAGGCAGCAG | i5 | GGATATCT |  |
| hV3F_MID8 | AATGATACGGCGACCACCGAGATCTACAC GACACCGT ACACTCTTTCCCTACACGACGCTCTTCCGATCT CCTACGGGAGGCAGCAG | i5 | GACACCGT |  |
| hV3F_MID9 | AATGATACGGCGACCACCGAGATCTACAC CTACTATA ACACTCTTTCCCTACACGACGCTCTTCCGATCT T CCTACGGGAGGCAGCAG | i5 | CTACTATA |  |
| hV3F_MID10 | AATGATACGGCGACCACCGAGATCTACAC CGTTACTA ACACTCTTTCCCTACACGACGCTCTTCCGATCT GT CCTACGGGAGGCAGCAG | i5 | CGTTACTA |  |
| hV4R_MID_C | CAAGCAGAAGACGGCATACGAGAT AGTAGCGT GTGACTGGAGTTCAGACGTGTGCTCTTCCGATCT TC GGACTACHVGGGTWTCTAAT | i7 |  | ACGCTACT |
| hV4R_MID_H | CAAGCAGAAGACGGCATACGAGAT GTCGCTCG GTGACTGGAGTTCAGACGTGTGCTCTTCCGATCT GGACTACHVGGGTWTCTAAT | i7 |  | CGAGCGAC |
| hV4R_MID_I | CAAGCAGAAGACGGCATACGAGAT GTCGTAGT GTGACTGGAGTTCAGACGTGTGCTCTTCCGATCT A GGACTACHVGGGTWTCTAAT | i7 |  | ACTACGAC |
| hV4R_MID_J | CAAGCAGAAGACGGCATACGAGAT TAGCAGAC GTGACTGGAGTTCAGACGTGTGCTCTTCCGATCT TC GGACTACHVGGGTWTCTAAT | i7 |  | GTCTGCTA |
| hV4R_MID_K | CAAGCAGAAGACGGCATACGAGAT TCATAGAC GTGACTGGAGTTCAGACGTGTGCTCTTCCGATCT CTA GGACTACHVGGGTWTCTAAT | i7 |  | GTCTATGA |
| hV4R_MID_L | CAAGCAGAAGACGGCATACGAGAT TCGCTATA GTGACTGGAGTTCAGACGTGTGCTCTTCCGATCT GATA GGACTACHVGGGTWTCTAAT | i7 |  | TATAGCGA |
| hV4R_MID_M | CAAGCAGAAGACGGCATACGAGAT AAGTCGAG GTGACTGGAGTTCAGACGTGTGCTCTTCCGATCT ACTCA GGACTACHVGGGTWTCTAAT | i7 |  | CTCGACTT |
| hV4R_MID_N | CAAGCAGAAGACGGCATACGAGAT ATACTTCG GTGACTGGAGTTCAGACGTGTGCTCTTCCGATCT TTCTCT GGACTACHVGGGTWTCTAAT | i7 |  | CGAAGTAT |
| hV4R_MID_O | CAAGCAGAAGACGGCATACGAGAT CATAGAGA GTGACTGGAGTTCAGACGTGTGCTCTTCCGATCT GGACTACHVGGGTWTCTAAT | i7 |  | TCTCTATG |
| hV4R_MID_P | CAAGCAGAAGACGGCATACGAGAT CGTAGATC GTGACTGGAGTTCAGACGTGTGCTCTTCCGATCT A GGACTACHVGGGTWTCTAAT | i7 |  | GATCTACG |
| hV4R_MID_Q | CAAGCAGAAGACGGCATACGAGAT GCGCACGT GTGACTGGAGTTCAGACGTGTGCTCTTCCGATCT TC GGACTACHVGGGTWTCTAAT | i7 |  | ACGTGCGC |
| hV4R_MID_R | CAAGCAGAAGACGGCATACGAGAT GGTACTAT GTGACTGGAGTTCAGACGTGTGCTCTTCCGATCT CTA GGACTACHVGGGTWTCTAAT | i7 |  | ATAGTACC |
| hV4R_MID_S | CAAGCAGAAGACGGCATACGAGAT TACGAGCA GTGACTGGAGTTCAGACGTGTGCTCTTCCGATCT GATA GGACTACHVGGGTWTCTAAT | i7 |  | TGCTCGTA |
| hV4R_MID_T | CAAGCAGAAGACGGCATACGAGAT TCAGCGTT GTGACTGGAGTTCAGACGTGTGCTCTTCCGATCT ACTCA GGACTACHVGGGTWTCTAAT | i7 |  | AACGCTGA |
| hV4R_MID_U | CAAGCAGAAGACGGCATACGAGAT AGCTGCTA GTGACTGGAGTTCAGACGTGTGCTCTTCCGATCT TTCTCT GGACTACHVGGGTWTCTAAT | i7 |  | TAGCAGCT |
| hV4R_MID_V | CAAGCAGAAGACGGCATACGAGAT CTCGTTAC GTGACTGGAGTTCAGACGTGTGCTCTTCCGATCT GGACTACHVGGGTWTCTAAT | i7 |  | GTAACGAG |
| hV4R_MID_W | CAAGCAGAAGACGGCATACGAGAT GTATACGC GTGACTGGAGTTCAGACGTGTGCTCTTCCGATCT A GGACTACHVGGGTWTCTAAT | i7 |  | GCGTATAC |
| hV4R_MID_X | CAAGCAGAAGACGGCATACGAGAT TCGCTACG GTGACTGGAGTTCAGACGTGTGCTCTTCCGATCT TC GGACTACHVGGGTWTCTAAT | i7 |  | CGTAGCGA |
