# Supplementary material for: Persistent reduction of Bifidobacterium longum in the infant gut microbiome in the first year of age following intrapartum penicillin prophylaxis for maternal GBS colonization
Source: Front Immunol. 2025 May 15;16:1540979. doi: 10.3389/fimmu.2025.1540979 (PMC12119681; doi:10.3389/fimmu.2025.1540979)
Supplement: Supplementary file 7 [file Table2.docx]

**Supplementary Table 2** List of used antibodies for flow cytometry analysis of peripheral blood

Fluorescence-labeled antibodies were used to examine the production of chemokines, cytokines and transcription factors produced by T helper cells, cytotoxic T cells and regulatory T cells in infant peripheral blood during the first days after birth.

| eBioscience Fixable Viability Dye eFluor 780 | Thermo Fisher Scientific, Waltham, USA |
| --- | --- |
| CD3 Monoclonal Antibody (UCHT1), Alexa Fluor 700,  eBioscience | Invitrogen, Thermo Fisher Scientific, Waltham, USA |
| Brilliant Violet 785 anti-human CD4 Antibody | BioLegend, San Diego, USA |
| Human CD25/IL-2R alpha Alexa Fluor 594-conjugated Antibody | R&D Systems, Minneapolis, USA |
| FOXP3 Monoclonal Antibody (PCH101), eFluor 660, eBioscience | Invitrogen, Thermo Fisher Scientific, Waltham, USA |
| BD Horizon BV421 Mouse Anti-Human RORγt | BD Biosciences, Becton, Dickinson and Company, Franklin Lakes, USA |
| Brilliant Violet 711 anti-human IL-17A Antibody | BioLegend, San Diego, USA |
| BD Horizon BV480 Mouse Anti-Human IFN-γ | BD Biosciences, Becton, Dickinson and Company, Franklin Lakes, USA |
| Brilliant Violet 650 anti-human IL-2 Antibody | BioLegend, San Diego, USA |
| IL-8 (1-77) (CXCL8) Monoclonal Antibody (8CH), PerCP-eFluor 710, eBioscience | Invitrogen, Thermo Fisher Scientific, Waltham, USA |
| IL-10 Monoclonal Antibody (JES3-9D7), Alexa Fluor 488,  eBioscience | Invitrogen, Thermo Fisher Scientific, Waltham, USA |
| TGF-beta Antibody (1D11.16.8) [DyLight 550] | Novus Biologicals, Centennial, USA |
