# Supplementary material for: Persistent reduction of Bifidobacterium longum in the infant gut microbiome in the first year of age following intrapartum penicillin prophylaxis for maternal GBS colonization
Source: Front Immunol. 2025 May 15;16:1540979. doi: 10.3389/fimmu.2025.1540979 (PMC12119681; doi:10.3389/fimmu.2025.1540979)
Supplement: Supplementary file 8 [file Table3.docx]

**Supplementary Table 3** Detailed characteristics of study participants at birth, at one month and one year after birth, respectively.

|  | **all** | **controls** | **IAP- exposed** | **p-value** | **test** |
| --- | --- | --- | --- | --- | --- |
| **n** | 48 | 26 | 22 |  |  |
| **at birth** |  |  |  |  |  |
| gestational age at birth [weeks], median (1.-3. quartile) | 40.1 (39.4-41) | 40.1 (39.48-41.08) | 40.1 (39.23-40.6) | 0.3728 | § |
| birth weight [grams], median (1.-3. quartile) | 3635 (3225-4100) | 3795 (3285-4137.5) | 3475 (3190-3822.5) | 0.1993 | § |
| SGA, n (%) | 5 (10.42) | 2 (7.69) | 3 (13.64) | 0.6492 | o |
| length at birth [cm], median (1.-3. quartile) | 53 (51-54.25) | 53 (52-55) | 52 (51-53.75) | 0.1618 | § |
| head circumference at birth [cm], median (1.-3. quartile) | 35 (34-36) | 35 (34-36) | 35 (34.25-36) | 0.7513 | § |
| female, n (%) | 31 (64.58) | 16 (61.54) | 15 (68.18) | 0.8598 | # |
| APGAR 1, median (1.-3. quartile) | 9 (8-9) | 9 (9-9) | 9 (8-9) | 0.1319 | § |
| APGAR 5, median (1.-3. quartile) | 10 (9-10) | 10 (9.25-10) | 10 (9-10) | 0.715 | § |
| APGAR 10, median (1.-3. quartile) | 10 (10-10) | 10 (10-10) | 10 (10-10) | 0.5283 | § |
| maternal age at birth [years], median (1.-3. quartile) | 31 (28-33) | 31 (28-32) | 32 (28-34) | 0.3654 | § |
| maternal BMI before pregnancy [kg/m²], median (1.-3. quartile) | 22.6 (20.93-26.36) | 23.53 (21.83-26.77) | 22.54 (19.89-25.87) | 0.3061 | § |
| maternal BMI at birth [kg/m²], median (1.-3. quartile) | 29.23 (25.97-31.11) | 29.23 (26.01-34.30) | 29.33 (26.02-30.64) | 0.4748 | § |
| maternal nutrition, n (%) | - | - | - | - |  |
| mixed diet | 45 (93.75) | 24 (92.31) | 21 (95.45) | 1 | o |
| vegetarian | 3 (6.25) | 2 (7.69) | 1 (4.55) | 1 | o |
| maternal antibiotics during pregnancy, n (%) | 10 (20.83) | 4 (15.38) | 6 (27.27) | 0.4777 | o |
| maternal cortisone during pregnancy, n (%) | 4 (8.51) | 1 (4) | 3 (13.64) | 0.3278 | o |
| maternal ethnicity, n (%) | - | - | - | - |  |
| Europe | 46 (95.83) | 26(100) | 20 (90.91) | 0.2048 | o |
| Asia | 2 (4.17) | 0 | 2 (9.09) | 0.2048 | o |
| smoking person at home, other than maternal, n (%) | 13 (27.08) | 7 (26.92) | 6 (27.27) | 1 | # |
| **at 1 month** |  |  |  |  |  |
| weight [grams], median (1.-3. quartile) | 4640 (4100; 5110) | 4770 (4080-5122.5) | 4600 (4180-4735) | 0.2953 | § |
| antibiotics child since birth, n (%) | 6 (12.5) | 3 (11.54) | 3 (13.64) | 1 | o |
| clinical EOS, n (%) | 3 (6.25) | 2 (7.69) | 1 (4.55) | 1 | o |
| blood culture positive sepsis, n (%) | 0 | 0 | 0 | - | - |
| hospitalization child postnatal, n (%) | 11 (23.40) | 8 (32) | 3 (13.64) | 0.2549 | # |
| breastmilk exclusively, n (%) | 27 (56.25) | 15 (57.69) | 12 (54.55) | 1 | # |
| formula exclusively, n (%) | 9 (18.75) | 5 (19.23) | 4 (18.18) | 1 | o |
| breastmilk and formula, n (%) | 12 (25) | 6 (23.08) | 6 (27.27) | 1 | # |
| probiotics since birth, n (%) | 3 (6.25) | 0 | 3 (13.64) | 0.08904 | o |
| maternal antibiotics since birth, n (%) | 2 (4.17) | 1 (3.85) | 1 (4.55) | 1 | o |
| maternal cortisone since birth, n (%) | 1 (2.13) | 1 (3.85) | 0 | 1 | o |
| maternal smoking, n (%) | 0 | 0 | 0 | - | - |
| smoking person at home, other than maternal, n (%) | 10 (20.83) | 7 (26.92) | 3 (13.64) | 0.3071 | o |
| **at 1 year** |  |  |  |  |  |
| weight [grams], median (1.-3. quartile) | 9720 (8647.5-10315) | 9620 (8577.5-10345) | 9760 (8922.5-10212.5) | 0.6674 | § |
| z-score for weight, median (1.-3. quartile) | 0.03(-0.65-0.54) | -0.31(-0.77-0.7) | 0.17(-0.19-0.52) | 0.5348 | § |
| BMI [kg/m²], median (1.-3. quartile) | 16.85 (16.02-17.64) | 16.82 (16.06-18.07) | 17 (15.98-17.47) | 0.5995 | § |
| z-score for BMI, median (1.-3. quartile) | 0.26(-0.32-0.74) | 0.19(-0.28-1.03) | 0.4(-0.34-0.67) | 0.5666 | § |
| obese, n (%) | 0 | 0 | 0 | - | - |
| overweight, n (%) | 3 (6.82) | 3 (11.54) | 0 | 0.2579 | o |
| breastmilk at one year, n (%) | 15 (31.25) | 9 (34.62) | 6 (27.27) | 0.8147 | # |
| if ever received breastmilk, completed month, median (1.-3. quartile) | 9.17 (4.6-12) | 8.27 (2.87-12) | 9.57 (6.17-12) | 0.7448 | § |
| introduction of complementary food, n (%) | 48 (100) | 26 (100) | 22 (100) | - | - |
| age of introduction of complementary food [month], median (1.-3. quartile) | 5 (4.5-6) | 5 (4.5-6) | 5 (4.63-5.75) | 0.759 | § |
| vegetarian nutrition child, n (%) | 3 (6.38) | 1 (4) | 2 (9.09) | 0.593 | o |
| medication child |  |  |  |  |  |
| antibiotic treatment after first month sampling, n (%) | 4 (8.51) | 1 (3.85) | 3 (14.29) | 0.3112 | o |
| antibiotic treatment since birth, n (%) | 10 (21.28) | 4 (15.38) | 6 (28.57) | 0.3064 | o |
| cortisone, n (%) | 6 (12.77) | 4 (15.38) | 2 (9.52) | 0.678 | o |
| probiotics child after first month sampling, n (%) | 3 (6.52) | 1 (3.85) | 2 (10) | 0.5718 | o |
| hospitalization child after first month sampling, n (%) | 7 (15.22) | 5 (20) | 2 (9.52) | 0.4285 | o |
| infection child, n (%) | - | - | - | - | - |
| bronchitis | 7 (14.58) | 4 (15.38) | 3 (13.64) | 1 | o |
| gastroenteritis | 9 (18.75) | 5 (19.23) | 4 (18.18) | 1 | o |
| flatulence | 11 (22.92) | 6 (23.08) | 5 (22.73) | 1 | # |
| diarrhea | 8 (16.67) | 5 (19.23) | 3 (13.64) | 0.7102 | o |
| diaper rash | 12 (25) | 8 (30.77) | 4 (18.18) | 0.5035 | # |
| atopic eczema | 8 (16.67) | 5 (19.23) | 3 (13.64) | 0.7102 | o |
| upper respiratory tract infection | 37 (78.72) | 20 (76.92) | 17 (80.95) | 1 | o |
| pneumonia | 0 | 0 | 0 | - | - |
| otitis media | 2 (4.26) | 0 | 2 (9.52) | 0.1943 | o |
| urinary tract infection | 2 (4.26) | 1 (3.85) | 1 (4.76) | 1 | o |
| allergy | 1 (2.08) | 1 (3.85) | 0 | 1 | o |
| autoimmune disease | 0 | 0 | 0 | - | - |
| children with siblings, n (%) | 22 (45.83) | 11 (42.31) | 11 (50) | 0.8086 | # |

Mann-Whitney-U-Test (§), Fisher's Exact Test (o), Pearson's Chi-squared test with Yates' continuity correction (#).

Abbreviations: SGA - small for gestational age, BMI - body mass index, EOS - early onset sepsis
